# Fatal Salivation from Bichloride of Mercury

**Published:** 1871-11

**Authors:** 


### Fatal Salivation from Bichloride of Mercury.

In a case which is fully reported in the *Lancet* for September 16, Dr. Meeres applied with a small camel's-hair brush a strong alcoholic solution of corrosive sublimate—eighty grains to the ounce—to the head of a child affected with tinea tonsurans. The application gave rise to no pain at the time, but during the ride home, in an open dog-cart, the child suffered severely. Shortly afterwards vomiting and purging came on. Salivation, accompanied by much swelling of the parotid and submaxillary glands, was first observed on the evening of the day after the application, and continued until death took place, apparently from prostration, on the morning of the fifth day.

The verdict of the coroner's jury was "that death was caused by poison from the application of a very strong preparation of bichloride of mercury, made to the head and neck by Dr. Meeres," and that "Dr. Meeres is very greatly to be blamed for having made the application."

The lotion applied was from a formula of Dr. Tilbury Fox, and has been used by him in a precisely similar manner in the same disease in very many instances, and this case is the first in which any untoward symptoms have been produced by it.—*Medical Times.*

